# Consumer anxiety: A scoping review and research agenda

**DOI:** 10.1371/journal.pone.0349021

**Published:** 2026-05-08

**Authors:** Mingfei Li, Shanshan Huang

**Affiliations:** 1 Research Center for Ecological and Cultural Tourism of Western Hubei, Hubei Minzu University, Enshi, Hubei, People’s Republic of China; 2 Hubei Minzu University, Enshi, Hubei, China; Tianjin University, CHINA

## Abstract

**Background:**

In the current vibrant and transforming era, consumers increasingly experience anxiety. Given its profound implications for consumers’ psychological well-being, decision-making, and consumption behaviors, understanding consumer anxiety (CA) has become a critical issue for both scholars and practitioners. Although existing studies have investigated CA across contexts, the findings remain both limited and fragmented.

**Objective:**

To provide a comprehensive and clearer picture of CA, this scoping review synthesizes the current state of the art in CA research, develops an integrative research framework, and outlines promising avenues for future inquiry.

**Method:**

Following the Systematic Reviews and Meta-Analyses (PRISMA) guidelines, this study searched Google Scholar, ScienceDirect, EBSCO, and the Social Science Citation Index for articles. After study selection and quality assessment, 60 articles focusing on CA were identified and included in the review.

**Results:**

Current research elucidates the antecedents and consequences of CA, as well as its mediating and moderating roles in consumer studies.

**Conclusion:**

On the basis of the reviewed findings, this study discusses the research gaps and thereby proposes promising avenues for future CA research. Moreover, several valuable insights for CA management are provided for practitioners in the marketplace.

## Introduction

For people in modern societies, anxiety is a critical and ubiquitous emotion. Recently, a survey reported that nearly half of Generation Z members and 40 percent of millennials feel anxious all or most of the time [1]. In the age of consumerism, consumer anxiety (CA) continually manifests in various consumption contexts [2]. Consumers tend to suffer more from anxiety than they did in the past because of global climate change, economic concerns, technological advancements, societal shifts, health concerns, and poor work/life balance [3]. CA not only harms consumers’ mental and physical health but also undermines relationships between consumers and firms, which was especially evident during and after the COVID-19 pandemic [4]. However, the literature has yet to present a holistic view of the role of CA in consumers’ lives.

This study aims to comprehensively review and synthesize the literature on CA, providing a structured and integrative understanding of its conceptualizations, antecedents, consequences, and mediating and moderating roles across diverse consumption contexts. Specifically, this review endeavors to (1) clarify how CA has been defined and operationalized in prior research; (2) categorize the key antecedents that elicit CA in consumption contexts; (3) consolidate the behavioral, cognitive, emotional, and attitudinal outcomes of CA; and (4) examine the boundary conditions under which CA promotes or suppresses consumer responses. By integrating fragmented knowledge and identifying theoretical gaps, this scoping review seeks to construct a coherent research network while outlining promising avenues for future CA research.

This review makes three contributions. First, this paper is among the first to integrate the current findings, providing an overview of CA research. In this study, a comprehensive research structure for CA is delineated based on empirical findings, including the antecedents, consequences, and mediating and moderating effects of CA in consumer contexts. Second, this paper discusses the current state of and promising avenues for CA research. Specifically, this study seeks to present the current state of the art in empirical CA research, thereby illuminating the boundaries of existing knowledge and identifying gaps in the CA literature. Third, we present valuable implications for practitioners in the marketplace regarding CA management. Managers and marketers can benefit from understanding the antecedents, outcomes, and coping strategies associated with CA to achieve a long-term competitive advantage.

The following sections first outline the methodology, including the search strategy, literature sources, article selection, quality assessment, and characteristics of the included articles. The review then examines the definitions and operationalizations of CA in the literature. Next, it synthesizes the primary empirical findings on the antecedents and consequences of CA, as well as its mediating and moderating roles. Finally, the review assesses the current state of CA research and concludes with directions for future research and practical implications for CA management.

## Methodology

**Fig 1:**
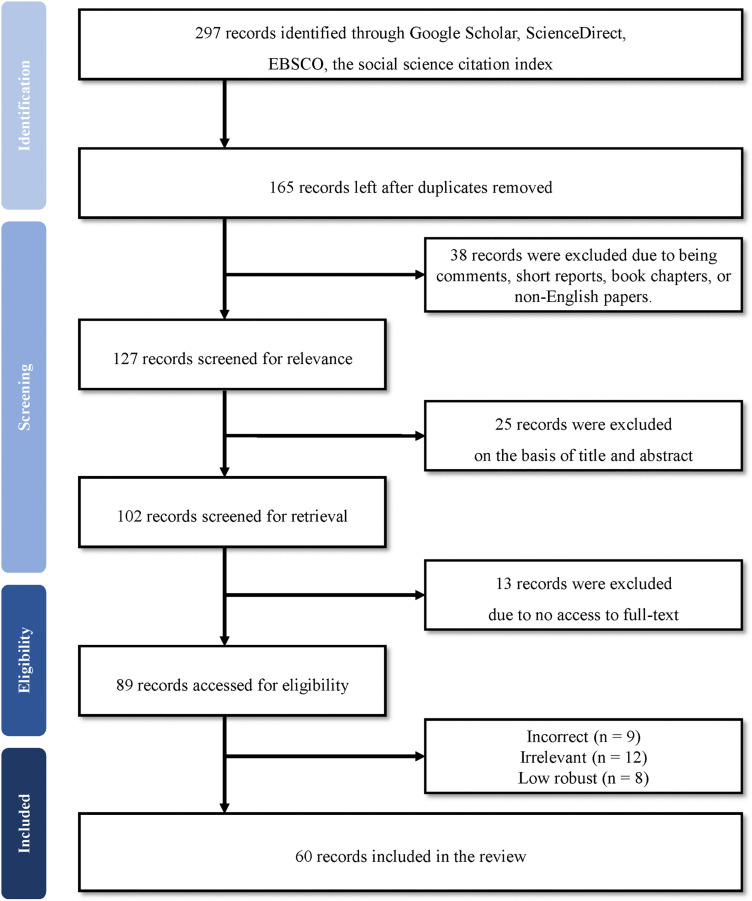
The PRISMA flow.

To capture and review prior CA research, this study followed the PRISMA flow [5,6] (see Fig 1). The PRISMA guidelines were developed as a protocol to minimize potential selection and publication bias. The primary objective of this scoping review is to synthesize the antecedents and consequences of CA and to present current evidence on its mediating and moderating roles. Thus, the PRISMA recommendations are suitable for identifying relevant literature and clearly presenting the current state of CA research. As the International Prospective Register of Systematic Reviews (PROSPERO) does not accommodate scoping reviews, no protocol was registered for this study. Nevertheless, this study rigorously followed the PRISMA guidelines [6] and employed a three-step procedure comprising literature search, selection, and analysis.

### Literature search: Strategy and results

To ensure a comprehensive search for relevant literature, this review includes all studies examining anxiety in various consumption contexts within its scope. An extensive keyword search was conducted in online databases, including Google Scholar, ScienceDirect, EBSCO, and the Social Science Citation Index. Specific search terms such as “consumer anxiety,” “customer anxiety,” “anxious consumers,” and “anxiety in consumption” were used. In addition, a Boolean phrase search approach was used [7]. For instance, the authors deployed the search query “consumer” AND “anxiety.” During this process, forward and backward searches were conducted to identify relevant articles from various fields and broaden the scope of the findings. The data were collected from 1995 to 2025 to understand the evolution of CA studies. Initially, 297 articles were extracted from the data sources (see Fig 1). After removing duplicates (n = 132), 165 records remained for the next stage.

### Literature selection: Screening and assessment

Recommended inclusion/exclusion criteria in the literature were employed to select the searched records [5,6]. First, only scientific articles written in English and published in peer-reviewed journals were included in the review pool [5]. Therefore, records such as comments, short reports, book chapters, and non-English papers were excluded (n = 38). Second, after 100% agreement was reached in the initial relevance check, 25 records were excluded based on the title and abstract. Among the remaining 102 articles, 13 articles were further excluded because of a lack of access to the full text. Then, 89 full-text publications were assessed for eligibility. Each article was assessed using criteria adopted by earlier systematic reviews reported in the literature [8], including the focus research question, research design, sampling and data analysis. Two authors independently evaluated all the screened articles. Content analysis was subsequently conducted to select eligible studies. Only empirical studies that included CA as a key variable in their framework were included. To ensure objective and comparable findings, articles were excluded (n = 29) if they were identified as incorrect (e.g., scope mismatch or research dissemination; n = 9), irrelevant (e.g., topical irrelevance or keyword traps; n = 12), or less robust (e.g., small sample sizes, poor measurement practices, low analytical rigor; n = 8). After the articles were screened according to the above inclusion and exclusion criteria, 60 articles were identified (see Fig 1).

### Literature analysis: Description of included works

**Fig 2:**
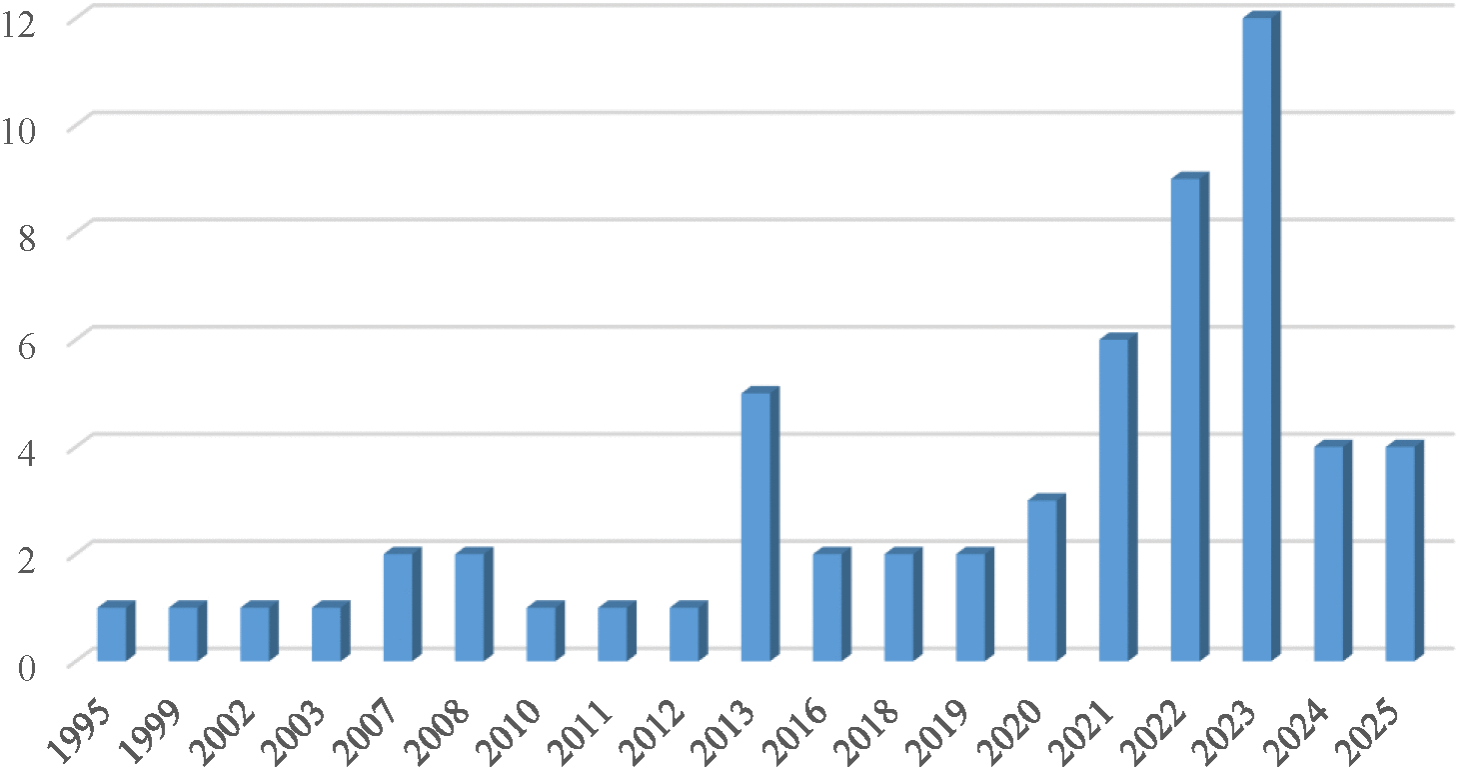
Number of publications from 1995 to 2025.

The reviewed articles indicate that research on CA has attracted increasing scholarly attention over the past three decades (see Fig 2). Among the identified papers, more than half of the CA studies (n = 35) were published within the most recent five-year period (2021–2025). Moreover, among the eligible papers, a total of 21 countries contributed to the CA literature. China produced the greatest number of CA articles (n = 16), followed by the United States (n = 15), the United Kingdom (n = 4), Korea (n = 3), and Spain, South Africa, India, Mexico, and Australia (each with n = 2). The remaining countries contributed one article each.

Considering the scholarly outlets of CA studies, the reviewed articles were published in 40 peer-reviewed journals (see Table 1). From a holistic perspective, CA has attracted the attention of multiple academic communities, spanning the domains of marketing, consumer behavior, psychology, information systems, tourism, and interdisciplinary studies. The marketing and consumer behavior domain has generated the greatest number of CA articles (60%). A total of nine papers were published in the *Journal of Retailing and Consumer Services*, followed by the *International Journal of Consumer Studies* and the *Journal of Business Research*, each of which published three papers. The *European Journal of Marketing, International Journal of Bank Marketing*, *Journal of Consumer Behaviour*, *Journal of Consumer Marketing*, *Journal of Marketing Communications*, *Journal of the Academy of Marketing Science*, *Current Psychology*, and *Information & Management* each of which published two papers. The remaining journals contributed one article each. Collectively, the breadth of journals in Table 1 highlights the interdisciplinary scope of CA research and its rapidly growing relevance across marketing, psychology, service management, and technology acceptance and adoption.

**Table 1:** Journals published in CA research.

Domains	Journals	Count
**Marketing & Consumer Behavior**	*Journal of Retailing and Consumer Services*	9
	*International Journal of Consumer Studies*	3
	*Journal of Business Research*	3
	*European Journal of Marketing*	2
	*International Journal of Bank Marketing*	2
	*Journal of Consumer Beha*v*iour*	2
	*Journal of Consumer Marketing*	2
	*Journal of Marketing Communications*	2
	*Journal of the Academy of Marketing Science*	2
	*Asia Pacific Journal of Marketing and Logistics*	1
	*Journal of Consumer Affairs*	1
	*Journal of Consumer Research*	1
	*Journal of Islamic Marketing*	1
	*Journal of Marketing*	1
	*Journal of Marketing Analytics*	1
	*Journal of Marketing Research*	1
	*Journal of Public Policy & Marketing*	1
	*Journal of Research in Interactive Marketing*	1
**Psychology**	*Current Psychology*	2
	*Acta Psychologica*	1
	*Appetite*	1
	*CyberPsychology & Behavior*	1
	*Depression and Anxiety*	1
	*Journal of Applied Social Psychology*	1
	*Journal of Personality and Social Psychology*	1
	*Psychological Reports*	1
	*Psychology & Marketing*	1
**Information Systems**	*Information & Management*	2
	*Computers in Human Behavior*	1
	*Decision Support Systems*	1
	*MIS Quarterly*	1
**Hospitality & Tourism**	*Journal of Hospitality and Tourism Management*	1
	*Current Issues in Tourism*	1
	*The Service Industries Journal*	1
	*Tourism Management*	1
**Ethics, Public Policy & Social Issues**	*Journal of Business Ethics*	1
	*Journal of Immigrant & Refugee Studies*	1
	*Journal of Public Policy & Marketing*	1
**Social Sciences**	*Cogent Business & Management*	1
	*Journal of System and Management Sciences*	1

## Main findings of consumer anxiety research

### Definition of consumer anxiety

**Fig 3:**
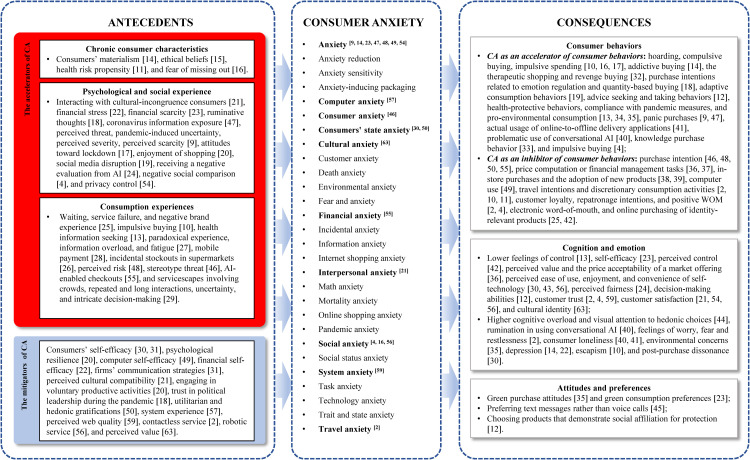
The antecedents and consequences of consumer anxiety. Note: In the “Consumer Anxiety” section, bolded operationalizations of CA with superscripts indicate that they serve a mediating role in the focal study.

Anxiety pertains to the experience of fear, apprehension, and discomfort within individuals in specific social situations [3]. In consumer studies, CA refers to consumers’ psychological responses to potential loss, threat, and risk in consumption contexts [9]. This review focuses on consumer anxiety across various consumption contexts, adopting a broad perspective that encompasses all forms of anxiety experienced by consumers. With respect to the conceptualization of CA, nearly one-third of CA studies employ anxiety directly. Two of the reviewed studies use the concept of consumer anxiety [10]. The other stream of research focuses on context-specific forms of anxiety, such as technology anxiety, social anxiety, and environmental anxiety, in various consumption contexts (see Fig 3). After the outbreak of the COVID-19 pandemic, health-related CA has received increased academic attention [11].

In psychology, anxiety is commonly conceptualized as comprising two core components: trait anxiety and state anxiety [12]. Similarly, existing consumer studies have investigated CA in two forms. In most theories of personality, trait anxiety is a stable consumer trait and a major component of personality [13]. In contrast, state anxiety is a transient reaction to stressful, situation-specific consumption contexts that fluctuates over time [13].

### Antecedents of consumer anxiety

#### Accelerators of consumer anxiety.

Prior research on the antecedents of CA can be broadly categorized into two types: accelerators and mitigators (see Fig 3). Most of the reviewed antecedent research has focused on the accelerators of CA. First, from a personality psychology perspective, enduring individual differences shape how consumers respond to potential threats in consumption environments. Specifically, consumer traits such as materialism [14], ethical beliefs [15], health risk propensity [11], and fear of missing out [16] can give rise to CA.

Second, consumers’ psychological and social experiences serve as critical antecedents of CA. In line with cognitive appraisal theory, CA arises when consumers perceive a consumption environment as uncertain, potentially threatening, and personally relevant. For instance, during the COVID-19 pandemic, exposure to extensive coronavirus-related information, perceived threat and severity, pandemic-induced uncertainty, and perceived scarcity contributed to heightened CA [9,17]. Similarly, rumination about uncertain future events [18] and disruptions in social media environments [19] reinforce threat-focused cognitive appraisals, thereby intensifying CA. Moreover, enjoyment of shopping exacerbates COVID-19-induced CA [20]. According to social comparison theory, negative social comparisons can intensify CA by highlighting perceived gaps in status [4]. Interactions with culturally incongruent consumers may similarly heighten self-consciousness, thereby increasing CA [21]. From the perspective of conservation of resources theory, financial stress and perceived financial scarcity represent direct threats to consumers’ economic resources, prompting heightened CA [22,23]. Furthermore, technological contexts can elicit CA through performance-related concerns. For instance, receiving negative evaluations from AI systems can heighten consumers’ apprehension, thereby intensifying CA [24].

Third, CA is often triggered by consumption experiences. Guided by cognitive appraisal theory, consumption experiences—such as prolonged waiting, service failures, negative brand encounters, or incidental stockouts—can provoke CA by disrupting the expectations of market offerings [25,26]. Moreover, CA may stem from the cognitive appraisal of contemporary consumption experiences. In technology-mediated environments, factors such as information overload, technological paradoxes, and digital fatigue can intensify CA by taxing consumers’ mental resources [27]. Even widely adopted financial innovations, such as mobile payment systems, can elicit CA [28]. Importantly, consumption behaviors themselves may sometimes trigger CA. For example, impulsive buying [10] and extensive health information seeking [13] increase CA. Insights from environmental psychology indicate that elements of the servicescape influence consumers’ emotional responses. Accordingly, service settings characterized by dense crowds, prolonged or repeated interactions, high uncertainty, or complex decision-making requirements can intensify CA [29].

#### Mitigators of consumer anxiety.

The remaining antecedent studies have investigated factors that mitigate CA. Specifically, consumers with higher technological self-efficacy tend to experience lower levels of CA when they purchase new technology products, in alignment with self-efficacy theory [30]. Similarly, financial self-efficacy serves as a protective resource, enabling consumers—such as African refugees in the southern United States—to effectively manage anxiety in unfamiliar financial environments [22]. Beyond individual capacities, firms’ communication strategies that improve process simulations can lower CA in new product adoption [31]. In cross-cultural customer-to-customer interactions, perceived cultural compatibility functions as a social**–**cognitive buffer, mitigating CA [21]. Consistent with resilience theory, consumers who engage in voluntary productive activities or who possess greater psychological resilience are less likely to experience heightened CA in response to pandemic-related threats [20]. In addition, trust in external authorities, such as political leadership, similarly reduced CA during the COVID-19 pandemic [18].

### Consequences of consumer anxiety

#### Consumer behaviors.

In the literature, CA has been conceptualized as a future-oriented emotional state that triggers psychological arousal and behavioral responses (see Fig 3). Our review reveals that the influence of CA on consumer behaviors is dualistic—it acts as both a promoter and an inhibitor—rather than serving as a uniform driver. This paradox can be understood through theoretical perspectives such as protection motivation theory and social identity theory, which elucidate how consumers cope with perceived risks and social uncertainty. Through the lens of protection motivation theory, CA functions as a threat signal that activates coping motivations in consumption contexts. These consumption behaviors are expected to mitigate psychological discomfort or restore the feeling of control, thereby stimulating protective or compensatory actions. For example, anxious consumers may resort to hoarding, compulsive buying, or impulsive spending as coping mechanisms to mitigate perceived uncertainty or restore emotional equilibrium [10,16,17]. Similarly, CA can heighten motivations for retail therapy, subsequently fueling “revenge buying” [32], or increase purchase intentions related to emotion regulation and quantity-based buying [9,18]. Within the broader context of ontological insecurity, CA can drive adaptive consumption, such as the purchase of essentials, entertainment, investment products, or self-care items that help restore psychological stability [19]. Furthermore, CA can motivate consumers to seek informational or social resources, thereby promoting advice-seeking behaviors [12] and knowledge purchases as coping strategies [33]. In risk-related consumption contexts, anxiety may further encourage protective or prosocial actions, including health-protective behaviors, compliance with pandemic measures, and pro-environmental consumption [13,34,35]. These findings suggest that when consumption is perceived as an effective coping strategy, CA activates protective motivations that translate into increased marketplace engagement.

Moreover, protection motivation theory helps explain the inhibitory effect of CA on consumer behavior. When perceived threat outweighs consumers’ coping appraisal or when purchasing is perceived as generating additional risk, consumers are more likely to withdraw and engage in avoidance behaviors. Specifically, CA related to numerical processing or financial decision-making can lead consumers to avoid price computation or financial management tasks [36,37]. Similarly, CA diminishes consumers’ willingness to adopt unfamiliar innovations (e.g., self-service technologies), discourages in-store purchases, and reduces the adoption of new products under conditions of high uncertainty [38,39]. In contexts such as pandemic-induced risk, heightened CA may further suppress travel intentions or other discretionary consumption activities [2,11]. This pattern indicates that when CA amplifies perceived threat without providing efficient coping strategies, consumers are likely to respond with behavioral inhibition.

Complementing these coping-based insights, social identity theory sheds light on the social dimension of anxious consumption. CA often stems from concerns about social evaluation, belonging, or identity threats, prompting proactive behaviors aimed at protecting or restoring consumers’ social standing. In these contexts, consumers may increase their engagement with socially visible consumption or digital platforms—including heightened use of social media and conversational AI systems—which can, in turn, trigger impulsive purchasing behaviors [4,40]. Social CA may also foster reliance on intermediated services, such as online-to-offline delivery applications, which enable consumers to sustain social participation while reducing the perceived risks of direct interpersonal interaction [41]. However, when CA impairs consumer confidence in social judgment—such as concerns about appearance or reputation—it may instead suppress behaviors such as electronic word-of-mouth sharing and online purchasing of identity-relevant products [25,42]. Furthermore, elevated CA can erode key indicators of customer–firm relationship quality, including brand loyalty, repatronage intentions, and positive word-of-mouth [2,4]. These findings suggest that when consumption functions as an effective coping mechanism or a means of restoring social identity, CA tends to enhance market engagement. Conversely, when CA heightens perceived risk, cognitive load, or concerns about social evaluation, it inhibits participation in consumer behaviors.

#### Cognition and emotion.

Anxiety also has pervasive effects on consumers’ cognitive and affective processes. In accordance with cognitive appraisal theory, anxious consumers frequently experience diminished perceived control and self-efficacy relative to their nonanxious counterparts [13,23]. When buying beauty products, social appearance-related CA erodes perceived control [42]. Consumers experiencing CA often perceive market offerings as less attractive or fair, a trend observed in both traditional pricing evaluations [36] and reactions to negative AI feedback [24]. Similarly, technology-infused service research reveals that CA diminishes the perceived ease of use of online platforms, reduces the enjoyment and convenience of mobile payment systems, and amplifies postpurchase dissonance [30,43]. Moreover, CA interferes with consumers’ decision-making capacities [12], reduces their trust in firms [2,4], and narrows their attentional focus, causing consumers to allocate disproportionate amounts of cognitive resources to hedonic options while experiencing cognitive overload [44]. This effect extends to interactions with AI systems, where CA increases rumination during the use of conversational AI [40]. Furthermore, CA heightens loneliness during the use of online-to-offline delivery applications [41] and conversational AI [40]. In addition, CA enhances depression in financial service settings [22] and escapism in retail contexts [10]. Notably, consumers with mortality-related anxiety are more likely to experience environmental concerns [35].

#### Attitudes and preferences.

Compared with nonanxious consumers, anxious consumers constitute a heterogeneous group with diverse attitudes and preferences. For example, CA positively affects green purchase attitudes [35] and green product preferences [23]. Moreover, since socially anxious consumers have neutral and distant relationships, they prefer text messages over voice calls [45]. In addition, anxious consumers tend to choose products that demonstrate social affiliation for protection [12].

### Mediating role of consumer anxiety

As summarized in Table 2, the extant research conceptualizes anxiety as a pivotal psychological mechanism through which situational stimuli translate into consumer outcomes [23,46,47]. Specifically, perceived risk in tourism contexts increases tourists’ anxiety, which in turn reduces their willingness to purchase package vacations [48]. Similarly, CA acts as the mechanism through which perceptions of financial scarcity influence consumption choices such as green purchasing [23]. In technology-infused service settings, CA helps explain the relationship between computer self-efficacy and

**Table 2:** Mediating and moderating roles of CA.

Study	CA as a		Research Context	Methodology			Main Findings
	Mediator	Moderator		Survey	Experiment	Other	
Blanco-González et al. (2023) [49]		√	Retailing	√			As the levels of CA increase, the impacts of company social responsibility on organizational legitimacy and revisit intention are strengthened.
Compeau and Higgins [50]	√		Technology-based services	√			CA helps explain the relationship between computer self-efficacy and usage.
Dabholkar and Bagozzi (2002) [51]		√	Technology-based self-service		√		Social CA strengthens the positive effects of perceived ease of use and fun on attitudes toward self-service technologies.
Dogra et al. (2023) [52]		√	Online tourism	√			Technical CA negatively moderates the effect of consumption value on purchase intention in online tourism settings.
Esmark Jones et al. (2020) [53]	√		Retailing		√		The negative relationship between privacy control and customer satisfaction is mediated by CA.
Ghazwani et al. [54]	√		Retailing		√		Among high-convenience consumers, AI-enabled checkouts lead to greater financial CA than do traditional forms of checkout, enhancing their purchase intentions.
Guo et al. (2024) [55]	√		Hotel room service, restaurant, beverage		√		In embarrassing service contexts, service robots (relative to human employees) lead to greater customer satisfaction, wherein social CA serves as a mediator.
Hackbarth et al. (2003) [56]	√		Information technology	√			Computer anxiety mediates the effect of system experience on ease of use.
Hsu et al. (2021) [57]		√	Mobile application service	√			As technology anxiety increases, the positive relationship between service failure severity and blame attribution weakens.
Hussain et al. (2023) [16]	√		Context-free	√			Fear of missing out increases consumers’ compulsive buying by enhancing CA.
Hwang and Kim (2007) [58]	√		e-Commerce	√			Users’ system anxiety mediates the effect of perceived web quality on trust.
Johnson and Grier (2013) [21]	√		Cross-cultural services		√		Intergroup CA mediates the relationship between perceived cultural compatibility and customer satisfaction in the context of cross-cultural services.
Keng and Liao (2013) [30]	√		Technology items consumption			√	State CA mediates the relationships between consumers’ chronic characteristics (i.e., trait anxiety and self-confidence) and postpurchase dissonance.
Khoa and Huynh (2022) [59]		√	Electronic commerce	√	√	√	CA weakens the effects of perceived mental benefits and online trust on electronic loyalty.
Lee and Yang (2013) [60]		√	Self-service	√			Technology anxiety enhances the effect of interpersonal service quality on retail patronage intention.
Lee et al. (2011) [46]	√		Financial service, car repair, car purchase		√		The marketplace stereotype threat effect is due to heightened CA.
Lee et al., (2022) [61]		√	Online shopping		√		Technical CA weakens the effects of AR-enhanced virtual try-on technologies’ attributes (perceived interactivity and perceived augmentation) on telepresence as well as the effect of telepresence on perceived hedonic value.
Li and Huang (2022) [2]	√		Hotel service		√		CA mediates the relationship between contactless service level and customer loyalty.
Lyu et al. (2023) [62]	√		Shopping center				In shopping centers, smartphone users’ utilitarian and hedonic gratifications significantly decrease their state anxiety, which further enhances their in-store purchase intention.
Maduku et al. (2023) [3]		√	Technology use			√	Technology anxiety attenuates the effects of consumers’ passion toward digital assistants on WOM intention and commitment to use.
Mitchell et al. (1999) [48]	√		Tourism		√		Travel anxiety mediates the relationship between perceived risk and intention to purchase a package holiday product.
Meng et al., (2025) [63]	√		Neo-Chinese fashion consumption		√		CA mediates the relationship between perceived value and cultural identity.
Mundel et al. (2023) [4]	√		Social media	√			Social anxiety and social media addiction mediates the influence of negative social comparison on impulsive buying.
Omar et al. (2021) [9]	√		Retailing	√			Pandemic-induced uncertainty, perceived severity, and perceived scarcity accelerate the aggravation of CA, which further increases the frequency of panic purchase behaviors.
Otero-López and Villardefrancos (2013) [14]	√		Context-free	√			CA mediates the impacts of the materialism dimensions (i.e., importance and success) on addictive buying.
Sherman et al. (2021) [47]	√		Retailing	√			COVID-19 information exposure affects panic buying directly and indirectly via CA.
Vlachos et al. (2010) [64]		√	Retailing	√			Attachment CA strengthens the relationships between consumer**–**firm emotional attachment and positive consumer outcomes (i.e., loyalty and WOM).
Yuan et al. (2022) [65]		√	AI-based services	√			Social CA strengthens the positive effects of AI assistant advantages on utilitarian and hedonic values.
Zhang et al. (2023) [23]	√		Retailing		√		The effect of financial scarcity on green consumption is mediated by CA.

usage [50]. Conversely, the utilitarian and hedonic gratification experienced by customers in a shopping center decreases state CA, which further enhances purchase intention [62]. Evidence from pandemic contexts clearly illustrates this mechanism: perceived uncertainty, severity, and scarcity [9], as well as exposure to coronavirus-related information [47], increased CA, which in turn led to panic purchasing behaviors. Beyond risk-related contexts, CA plays a key role in the marketplace stereotype threat effect, which facilitates consumers’ reluctance to purchase from outgroup members [46]. Similarly, negative social comparison can heighten CA about one’s social standing, which then contributes to impulsive buying behaviors [4]. The dimensions of materialism (i.e., importance and success) also increase CA, thereby indirectly promoting addictive buying behaviors [14]. Fear of missing out increases consumers’ compulsive buying behavior by enhancing CA [16]. Finally, CA mediates the influence of the technological or situational features of consumption environments. Among high-convenience consumers, AI-enabled checkouts lead to greater financial CA than traditional forms of checkout do, thus enhancing consumer purchase intentions [54].

Anxiety has also been proposed as an underlying process in the consumer experience [2]. Specifically, CA affects the link between chronic consumer characteristics and postpurchase dissonance [30]. In the context of technology use, computer-related CA fully transmits the effect of system experience on ease of use [56]. Moreover, system-related CA mediates the effect of perceived web quality on e-trust in online settings [58]. CA also serves as a mediator in retail contexts, transmitting the negative effect of privacy control on customer satisfaction [53]. Health-related CA helps explain the relationship between pandemic-induced service practices (i.e., contactless services) and consumer loyalty [2]. In embarrassing service contexts, service robots (compared with human employees) lead to greater customer satisfaction, with social CA serving as a mediator [55]. When Neo-Chinese fashion consumer behaviors are explored, researchers have indicated that CA mediates the relationship between perceived value and cultural identity [63]. In addition, in cross-cultural service settings, CA also mediates the relationship between perceived cultural compatibility and customer satisfaction [21].

### Moderating role of consumer anxiety

In consumer studies, CA has diverse moderating effects (see Table 2). In technology-based services, CA enhances the effect of interpersonal service quality on retail patronage intention [60]. Social CA strengthens the positive effects of perceived ease of use and fun on attitudes toward self-service technologies [51]. Moreover, CA amplifies the positive effects of AI assistant advantages on utilitarian and hedonic values [65]. With respect to service relationships, CA strengthens the relationship between consumer–firm emotional attachments and positive consumer outcomes [64]. As the level of CA increases, the effects of corporate social responsibility on organizational legitimacy and revisit intentions also become stronger [49].

However, amid the growth of CA, the impact of organizational legitimacy on revisit intentions is attenuated [49]. Similarly, the positive relationship between service failure severity and blame attribution is also weakened [57]. Moreover, CA dampens the effects of perceived mental benefits and online trust on electronic loyalty [59]. CA also restrains the effects of consumers’ passion for digital assistants on their WOM intention and commitment to use digital assistants [3]. In addition, CA negatively moderates the effect of consumption value on purchase intention in online tourism settings [52]. It also mitigates the effects of AR-enhanced virtual try-on technology attributes—perceived interactivity and perceived augmentation—on telepresence, as well as the relationship between telepresence and perceived hedonic value [61].

## Discussion and conclusion

### Theoretical implications and future research avenues

#### Theoretical lens in consumer anxiety research.

Prior research has investigated CA through several theoretical lenses, including anxiety/uncertainty management theory, affect-as-information theory, ontological security theory [19], and theories on control and coping [2,53]. Although these theoretical explorations have added insightful contributions to the CA literature, additional theories could be introduced to examine the various roles of anxiety in consumers’ lives. In examining CA from social perspectives, identity-based theories can be considered [55]. For example, social identity theory can help us understand how CA emerges within consumption environments involving social judgments and group exclusion [3]. Moreover, anxiety, as part of the motivational system [61], can govern consumption behavior; therefore, future research could use protection motivation theory to investigate the driving effect of CA on consumer behavior. Furthermore, vulnerable consumers have attracted increasing academic attention [5]. According to stereotype threat theory [46], some consumption experiences may activate negative stereotypes among vulnerable consumers, thereby accelerating CA. In addition, regulatory focus theory could help explain the behaviors of anxious consumers because anxiety may be related to a prevention focus in which vigilance is maintained [62]. Future studies could also empirically examine the linkages among regulatory focus, CA, and consumer behavior.

#### Research directions for consumer anxiety.

**Outcomes of consumer anxiety:** This scoping review revealed that more than half of CA studies have explored the consequences of CA. Nevertheless, several critical outcome variables have not yet been examined. For instance, the current literature shows that CA promotes certain consumer behaviors while inhibiting others. Therefore, further academic efforts are needed to elucidate the dual effects of CA on consumer behavior. For instance, future research could examine the factors that moderate the relationship between CA and distinct consumption patterns, such as impulsive consumption versus avoidant consumption. Moreover, the outcomes of CA in service recovery have not yet been examined. Investigating the effects of CA on different recovery strategies may provide detailed insights into consumers’ complex emotional reactions to service failures. Considering that CA entails economic, temporal, and psychological costs for consumers, future research could further examine the effect of CA on consumer well-being, and vice versa. With the rapid advancement of STARA technologies (smart technology, artificial intelligence, robotics, and algorithms), exploring the connections between CA, technology dependence, and human**–**AI reliance imbalance is a key avenue for future research. Additionally, further investigation is needed to clarify the ethical consequences of CA-driven technology adoption, such as dehumanization, moral disengagement, and moral decoupling.

**Antecedents of consumer anxiety:** The current knowledge network of CA highlights several promising avenues for future research, particularly its antecedents. For example, multiple studies have examined how individual predispositions act as accelerators of CA. Consumers may vary in their susceptibility or resilience to anxiety. However, several important chronic traits have not yet been considered (e.g., sociability, optimism, and compassion) [2]. Moreover, more detailed insights into the relationships between CA and demographic characteristics are needed. For example, scholars have suggested that young consumers exhibit high susceptibility to anxiety. In addition, STARA technologies (e.g., service robots and intelligent personal assistants) are being increasingly deployed by firms, shaping the ways in which consumers receive market offerings [5]. The increasing deployment of STARA technologies may engender new accelerators of CA [66], such as AI decision aids, algorithmic opacity, autonomy loss, unconsented surveillance, and privacy breaches.

Our review found that only thirteen studies have examined factors that alleviate CA, thus highlighting the need for further research on its mitigators. Some studies have discussed the social impacts of CA and established its significance for consumer well-being [2]. If anxiety is considered a negative indicator of quality of life, investigating ways to reduce CA for improved consumer well-being is warranted. Moreover, more attention should be given to the effects of marketing tactics on CA in a transforming era. Scholars have argued that loneliness predicts CA [67]. Thus, loneliness-reducing services or products may have the potential to hold CA. In addition, firms’ marketing tactics can both engender and manage CA. However, the roles of anxiety-provoking and anxiety-alleviating marketing tactics in CA have yet been adequately examined.

The reviewed studies have also established CA as a central intervening mechanism bridging diverse precursors and consumer outcomes (see Fig 3). Despite these theoretical advances, several promising research avenues remain underexplored. Future studies should move beyond single-path models toward dynamic, multistage frameworks that capture how different forms of CA transmit, amplify, or redirect the effects of consumption-related stimuli across contexts and over time. Our scoping review demonstrates that CA functions as a context-sensitive boundary condition (see Table 2). However, research examining the conditions under which CA functions as an amplifier versus an inhibitor of consumer reactions remains limited. Moreover, CA may interact with personality traits, further influencing key customer outcomes, such as consumption evaluation and customer–firm relationships. Hence, future research could examine how CA shapes the effects of individual differences, thereby offering a more nuanced understanding of consumer responses.

#### Operationalizations of consumer anxiety.

The operationalizations of CA take different forms in the reviewed studies. Most reviewed studies have treated CA as a unitary construct and explored state anxiety emerging from different consumption contexts [9,62,60]. However, the literature has acknowledged that trait anxiety is a major component of personality [30]. Hence, it is important to examine how both transient and chronic anxiety influence consumer behavior to uncover the diverse roles of CA. Furthermore, most reviewed studies treat CA as a homogeneous emotional state and often rely on generalized anxiety scales [47], although some studies use more specialized measures such as computer anxiety or pandemic-related travel anxiety. Employing context-specific measures of CA improves construct validity, reliability, and predictive power, yielding clearer and more precise insights into the phenomenon. Accordingly, the use of tailored CA measures is recommended to better capture its diverse effects and spillover influences on consumer decision-making.

#### Methodologies in consumer anxiety research.

With respect to methodology, the majority of CA studies (n = 37) have employed a survey to collect consumer data [27]. While the strengths and limitations of survey methodology have been recognized, future research on CA should prioritize experimental designs, with particular emphasis on field experiments, to strengthen causal inference and enhance the ecological validity of the findings. An increasing number of consumer researchers have asserted that CA should be treated as a transient emotion [13]. However, our review indicates that existing studies rarely employ research designs capable of capturing the dynamic fluctuations of CA, revealing a critical methodological gap in the literature. Compared with retrospective self-report measures, field experiments allow researchers to assess anxiety more precisely and establish stronger causal inferences, thereby generating deeper insights into CA. Moreover, longitudinal research helps assess whether CA-induced impulsive consumption evolves into compulsive consumption or subsequent avoidance [4]. Future research could employ longitudinal designs to track the habituation of CA and examine how it influences consumer behavior over time. In addition, as the CA literature evolves, additional empirical studies examining specific topics or the strength and direction of relationships between variables will provide sufficient evidence for calculating effect sizes in meta-analyses, representing a promising avenue for future research.

### Practical implications and managerial guidelines

Although the population of anxious consumers is increasing irreversibly, CA is a manageable phenomenon [62]. Consumers can cope with CA by engaging in various activities [20]. Hence, this review also provides some insightful implications for practitioners. First, better informing consumers may help curb CA. Anxious consumers are more likely to be suspicious of their decisions than nonanxious consumers are [2]. Thus, customer education practices can assuage CA by offering real-time and tailored information that anxious consumers need. Second, anxious consumers tend to prefer human customer service agents; thus, direct and in-person contacts should be made available, especially in anxiety-laden and technology-intensive contexts (e.g., medical and financial services) [24,28]. Third, providing consumers with various communication channels (e.g., apps and intelligent online assistants) may assuage CA by offering them instant connections and interactions. Fourth, firms should empower anxious consumers with control. Customer empowerment can increase customer competence and the locus of control [54], which can alleviate CA. Finally, marketers could develop more effective customer experience management programs to mitigate CA. For example, quality of life marketing and relationship marketing practices can benefit firms in the long term [2]. Despite the detrimental effects of CA on consumers’ psychological health, firms may employ marketing tactics that induce CA to stimulate sales. Thus, the manipulation of CA might lead to moral issues in business practices [15]. Thus, firms planning to establish an ethical image in the marketplace should prohibit the use of sales techniques that can induce CA.

## Conclusion

Given the prevalence and significance of anxiety in consumers’ lives, CA has attracted increasing scholarly attention in recent decades. This scoping review synthesizes the current state of knowledge on CA, offering important implications for both academia and practice. Our analysis demonstrates that CA research has grown rapidly—particularly in the past five years—and spans a broad range of themes, including its antecedents, outcomes, mediating mechanisms, and moderating conditions. By integrating these fragmented insights, this review provides a more comprehensive understanding of CA and identifies several underexplored areas that warrant further investigation. Nevertheless, this study has several limitations inherent to scoping reviews. First, the descriptive and integrative nature of this approach limits the ability to establish causal relationships among CA-related constructs. Future research employing meta-analytic techniques could provide more robust evidence regarding the magnitude and boundary conditions of these effects. Second, although this review follows established PRISMA guidelines to ensure transparency and rigor, potential selection bias cannot be entirely ruled out. Decisions related to database selection, keyword specification, and inclusion criteria may have influenced the final sample of studies. Additionally, the unavailability of full text for certain articles may have constrained the comprehensiveness of the review. Taken together, these limitations highlight the need for continued research to expand and refine the nomological network of CA. Accordingly, this review outlines several promising directions for future research. This work aims to stimulate further scholarly attention to CA, ultimately contributing to the mitigation of its negative consequences while harnessing its potential positive effects in consumer contexts.

## Supporting information

S1 File PRISMA flow, data Analysis, and checklist. (ZIP)
